# Supplementary material for: Predation and fragmentation portrayed in the statistical structure of prey time series
Source: BMC Ecol. 2009 May 6;9:10. doi: 10.1186/1472-6785-9-10 (PMC2689204; doi:10.1186/1472-6785-9-10)
Supplement: Additional file 2 — Voles and related classes ODDox Documentation. ODDox documentation of the agent-based model (ALMaSS) applied by Hendrichsen et al. The documentation is started by activating main.html. [file 1472-6785-9-10-S2.zip › Vole_ODDox/_population_manager_8h.html]

ALMaSS ODDox: PopulationManager.h File Reference

- Main Page
- Related Pages
- Classes
- Files

# PopulationManager.h File Reference

---

## Detailed Description

**PopulationManager.h This is the header file for the population manager and associated classes**   

by Chris J. Topping   
Version of 23rd July 2003   
All rights reserved.   
  
With additions as noted in:   
January 2008   
Doxygen formatted comments in May 2008   

|  |  |
| --- | --- |
|  |  |
| Classes | |
| class | AnimalPosition |
|  | A class defining an animals position. More... |
| struct | IntArray100 |
|  | A struct of 100 ints. More... |
| class | Population\_Manager |
|  | Base class for all population managers. More... |
| class | probe\_data |
|  | Data structure to hold & output probe data probe data is designed to be used to return the number of objects in a given area or areas in specific element or vegetation types or farms. More... |
| struct | rectangle |
|  | A struct defining two x,y coordinate sets. More... |
| class | RoeDeerInfo |
|  | Part of the basic ALMaSS system (obselete). More... |
| class | TALMaSSObject |
|  | The base class of all ALMaSS objects requiring Step code. More... |
| class | TAnimal |
|  | The base class for all ALMaSS animal classes. More... |
| Defines | |
| #define | \_\_lgridsize   39 |
| Typedefs | |
| typedef char \* | AnsiString |
| typedef vector< TAnimal \* > | TListOfAnimals |

---

## Define Documentation

#define \_\_lgridsize   39

Referenced by Population\_Manager::LamdaBirth(), and Population\_Manager::LamdaDeath().

---

## Typedef Documentation

typedef char\* AnsiString

Added in Jan 08 to facilitate ecological theory simulations

typedef vector< TAnimal \* > TListOfAnimals

---

Generated on Thu Jan 22 14:13:45 2009 for ALMaSS ODDox by 
 1.5.6
